# New bridging eco-acoustic indices inspired by deep neural networks for fine-grained bird vocalization recognition across diurnal cycles

**DOI:** 10.1371/journal.pone.0328098

**Published:** 2025-10-17

**Authors:** Lianglian Gu, Wei Li, Guangzhi Di, Danju Lv, Yan Zhang, Yueyun Yu, Ziqian Wang

**Affiliations:** 1 School of Big Data and Intelligent Engineering, Southwest Forestry University, Kunming, Yunnan, China; 2 Southwest Forestry University, Kunming, Yunnan, China; 3 School of Science, Southwest Forestry University, Southwest Forestry University, Kunming, Yunnan, China; Tongji University, CHINA

## Abstract

Revealing difference in bird vocalization changes from the perspectives of song recognition and acoustic indices has become a hot topic and challenge in recent ecological landscape research. This paper proposes a fine-grained (Dawn, noon, night) bird vocalization recognition framework based on a two-layer deep network to identify the same species’ bird vocalization at different times of the day. Additionally, a new acoustic index method, the Log-Mel Acoustic Complexity Index (Log-Mel ACI), is introduced to explore the differences in bird vocalization of the same species throughout the day. The results of two-layer deep network showed significant separability of the bird vocalization of the same species at dawn, noon, and night based on Log-Mel spectrum. Furthermore, it was found that the improved ACI based on Log-Mel exhibits better circadian rhythmic performance than the traditional ACI, being highest at dawn, followed by night, and lowest at noon. These findings demonstrate that Log-Mel is effective in both deep network recognition and ACI calculation.

## Introduction

Birds are a crucial component of ecosystems, and their behavior and communication deeply influence ecological dynamics. Bird vocalization is not only a fundamental means of communication for birds but also a critical strategy for responding to environmental changes, reproduction, and territory defense [1]. With the severity of ecological degradation and climate fluctuations, in-depth research into avian communication behaviors is crucial for maintaining biodiversity and interpreting ecological changes. Research shows that bird vocalization patterns change significantly at different times of the day, with many of the birds singing most actively at dawn and then gradually weakening after sunrise [2–6]. Studies have shown that bird vocalizations are dynamic traits that vary with the time of day and season [6]. Therefore, bird vocalization has a certain distinguishability at dawn, noon and night. By analyzing the differences in the sounds of birds at dawn, noon and night, researchers can reveal their activity patterns and environmental response mechanisms. This information is essential for developing effective bird detection programs [7].

Acoustic Complexity Index (ACI) is an important eco-acoustic index used to quantify the complexity of soundscape and biodiversity. It was first proposed by Pieretti et al. in 2011 [8]. Since then, it has been widely used in the study of ecological monitoring and environmental assessment [9,10]. ACI mainly measures the biodiversity of ecosystems by analyzing changes in sound intensity. Although traditionally used to compare sound complexity between different ecological environments, studies have shown that ACI can also reflect temporal variations in acoustic environments to a certain extent [30,31], most of these applications are based on long-duration environmental soundscapes. In contrast, this study pioneers the use of ACI on short-duration, individual bird vocalizations. This represents a novel application of ACI at a finer temporal scale, enabling the analysis of vocal behavior differences within the same species across different times of the day. However, the traditional ACI calculation method is mainly used to compare the sound complexity in different ecological environments, and there are some limitations in the detection of the same species’ vocalization characteristics in different time periods. Because ACI mainly depends on the change of sound intensity, it is difficult to capture the subtle temporal dynamics of bird vocalization in a day, which leads to its lack of fine analysis ability in the study of bird biological rhythm [10]. Therefore, improving the calculation method of ACI to make it more suitable for the time dynamic analysis of bird vocalization has become an important direction of current ecological acoustic research.

In recent years, deep learning technology has made significant progress in the field of bird song recognition, providing a powerful tool for automated bird population monitoring and behavior analysis. For example, the use of Convolutional Neural Networks (CNNs) [11,12] and Residual Networks (ResNet18) [13] significantly improves the recognition accuracy and efficiency. Especially, fine-grained recognition technology, which focuses on the sound characteristics of the same species at different times and under different environmental conditions [14], which allows researchers to gain a deeper understanding of bird behavior patterns and ecological habits. Existing research on bird vocalization recognition mainly focuses on inter-species recognition and has made significant progress. In 1998, dynamic time warping and Gadwall hidden Markov models were applied to achieve automatic recognition of bird vocalization elements, particularly in continuous recordings, laying the foundation for automated bird vocalization processing [15]. In recent years, the use of deep learning technology in bird vocalization recognition has become increasingly widespread. For instance, in 2015, the introduction of the deep residual learning (ResNet) model by He Kaiming. led to a significant breakthrough in image recognition, and this approach was later applied to audio signal classification tasks, substantially improving classification accuracy [13]. In 2021, a study proposed a bird vocalization recognition method based on multi-feature fusion, which addressed the challenge of complex and diverse audio backgrounds in bird vocalization datasets [16], which introduced the acoustic feature Chroma, combining it with Log-Mel Spectrogram and Mel Frequency Cepstral Coefficients (MFCC), and designed a model that integrated 3D-CNN and LSTM to enhance sensitivity to temporal changes in bird vocalization. In 2023, a study conducted a comparative analysis of different deep learning models for bird vocalization recognition, and further progress was made. [17]. This study analyzes three models - Artificial Neural Network (ANN), Convolutional Neural Network (CNN) and Recurrent Neural Network-Long Short-Term Memory (RNN-LSTM) - and uses Mel spectrum to extract MFCC as input data. The results show that RNN-LSTM provides the highest accuracy, followed by CNN and ANN, highlighting the effectiveness of deep learning in this field. Despite these advancements, most existing research has primarily focused on recognition tasks between different bird species [18–21], with relatively few studies addressing the fine-grained recognition of the same species’ songs at different times of the day.

This study explores the song patterns of birds in different time periods from two different perspectives : fine-grained recognition network and Log-Mel ACI acoustic indicators. Fine-grained recognition refers to a two-layer ResNet network. The first layer performs interspecific recognition of multiple birds, and the second layer performs recognition of individual birds at different time periods. The Log-Mel acoustic complexity index (Log-Mel ACI), by introducing the Log-Mel filter bank into the ACI calculation, enhances the applicability of ACI in the analysis of the dynamic changes of bird song time. The Log-Mel filter bank simulates human auditory perception and maps the linear frequency to the Mel scale, thereby improving the perception of biological acoustic features. Subsequently, the logarithm of the Mel spectrum was taken and the ACI value was calculated to more accurately characterize the song patterns of birds in the morning, noon and night. The contributions of this paper are as follows:

1. The Log-Mel Acoustic Complexity Index (Log-Mel ACI) is proposed by integrating the Log-Mel filter bank with the Acoustic Complexity Index (ACI) for the first time. This method is used to analyze and distinguish bird vocalization at different times of the day.
2. In this study, a two-layer deep neural network framework was constructed to not only classify different bird vocalization, but also fine-grainedly classify the vocalization of the same bird species at dawn, noon and night.
3. The relationship between fine-grained recognition and ACI calculation is established by Log-Mel, which shows the effectiveness of Log-Mel in deep network recognition and ACI calculation.

## Datasets and methods

**Fig 1:**
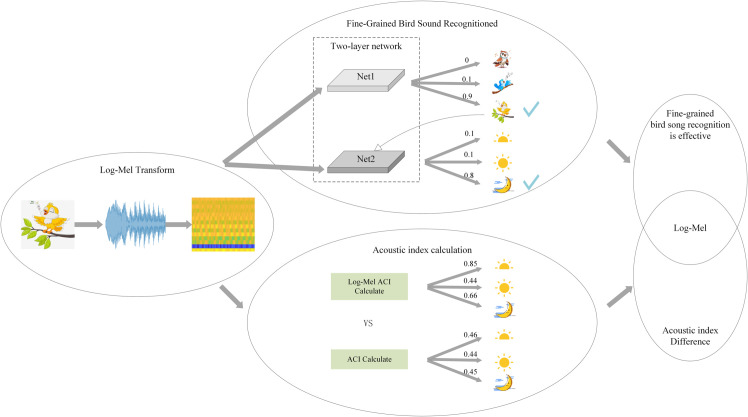
Log-Mel for double layer networks and acoustic index ACI.

The main research content of this study is shown in Fig 1, which illustrates the design of a two-layer network structure for fine-grained bird vocalization recognition and Log-Mel Acoustic Complexity Index (ACI) calculation. On the one hand, the study designed a two-layer network structure, in which the first layer (Net1) is used to distinguish different bird species, and then each single class in the first layer is placed in the second layer (Net2), that is, the same bird species is classified at different time periods (dawn, noon and night). Achieve the final fine-grained recognition. On the other hand, Log-Mel transform is used to extract the key features of bird vocalization and calculate ACI based on these features. Different from the traditional ACI calculation method that directly analyzes the original audio signal, Log-Mel ACI enhances the expression ability of bioacoustic signals through frequency domain conversion. Firstly, Log-Mel transform is performed on the audio data, and then the acoustic complexity index (ACI) is calculated. Finally, it is used to analyze the ecological characteristics of bird vocalization.

**Fig 2:**
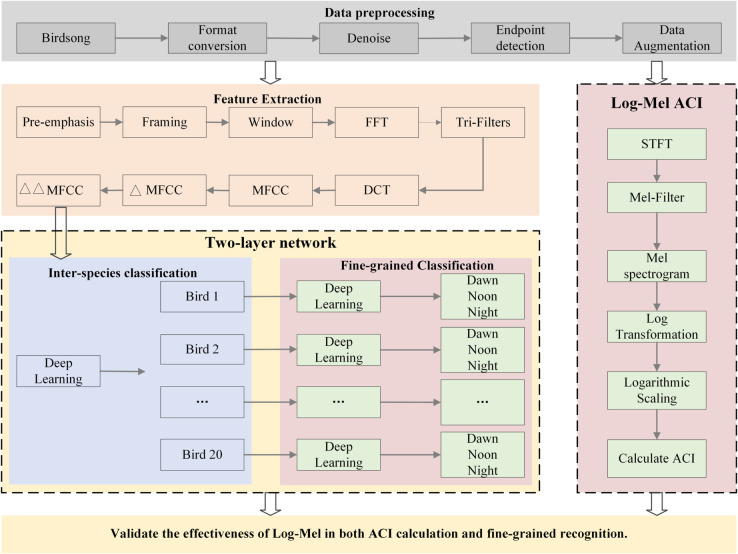
The overall technical roadmap of the study.

The overall technical roadmap of the study is shown in Fig 2. The entire research process is divided into the following main steps: data preprocessing, feature extraction, two-layer fine-grained recognition, and Log-Mel ACI calculation. Initially, bird vocalization data preprocessing steps include format conversion, denoising, endpoint detection, and data enhancement. Then, MFCC, first-order MFCC and second-order MFCC features are extracted by Log-Mel transform, time domain and frequency domain methods. A two-layer fine-grained recognition model was constructed : the first layer (Net1) was used to distinguish different bird species, and the second layer (Net2) further classified the vocalization of the same species according to time (dawn, noon, night). In addition, ACI is calculated based on Log-Mel to quantify the acoustic complexity of bird vocalization in different time periods, so as to reveal its time variation law. Compared with the traditional ACI calculation method, Log-Mel ACI can more effectively capture the ecological acoustic differences. Finally, the study verifies the effectiveness of Log-Mel in fine-grained identification and ACI calculation, indicating that the method has important application value in ecological monitoring and bird behavior analysis.

### Datasets and data preprocessing

This study selected audio of 20 different bird species at dawn, noon, and night, as shown in Table 1, The data source is the xeno-canto website, which is a global bird vocalizations collection and sharing platform, including recordings from different ecological regions, with detailed geographic information, providing a wealth of acoustic samples for this study. The selection of species is mainly based on three criteria : First, considering the ecological significance, birds with obvious circadian rhythms are selected to study the call patterns at different time periods ; secondly, considering the availability of data, species with sufficient recording quantity and high quality are selected to ensure the reliability of data. Finally, geographical diversity is considered to cover different ecological environments and improve the generalization ability of research methods. In terms of recording quality control, this study only uses audio with clear recording, high signal-to-noise ratio and low background noise. Finally, we downloaded a total of 6065 recorded data of different birds at dawn (4 : 00-8 : 00), noon (12 : 00-16 : 00) and night ( 18 : 00-22 : 00 ) to analyze the song rules of the same species in different time periods.

**Table 1:** 20 kinds of bird vocalization data at different time stages.

Bird Name	Family	Dawn	Noon	Night
*Cyanocittacristata*	Corvidae	109	73	14
*Pipiloerythrophthalmus*	Passerellidae	255	70	68
*Cardueliscarduelis*	Fringillidae	92	50	47
*Marecastrepera*	Anatidae	91	55	68
*Larusargentatus*	Laridae	260	85	46
*Colaptesauratus*	Picidae	117	49	25
*Agelaiusphoeniceus*	Icteridae	259	83	106
*Cardinaliscardinalis*	Cardinalidae	109	73	41
*Troglodytesaedon*	Troglodytidae	558	184	82
*Passerdomesticus*	Passeridae	749	413	228
*Zonotrichialeucophrys*	Passerellidae	121	61	33
*Icterusgalbula*	Icteridae	102	27	23
*Certhiaamericana*	Certhiidae	103	57	18
*Myiarchuscrinitus*	Tyrannidae	72	34	41
*Laniusexcubitor*	Laniidae	88	41	28
*Passerinacyanea*	Fringillidae	63	20	13
*Seiurusaurocapilla*	Parulidae	70	36	22
*Haemorhouspurpureus*	Fringillidae	83	39	21
*Pirangarubra*	Cardinalidae	103	31	45
*Contopussordidulus*	Tyrannidae	118	55	38

**Fig 3:**
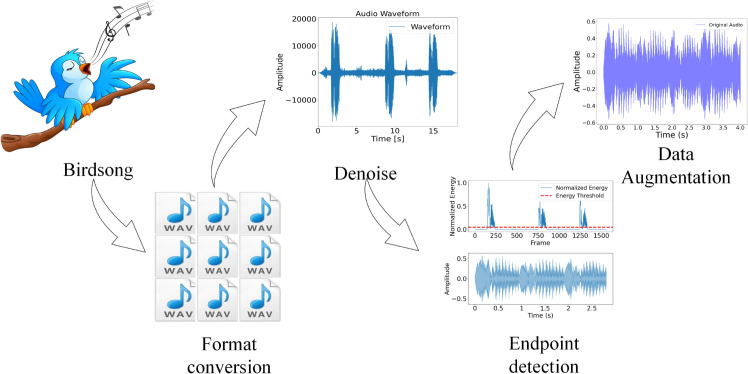
The process of bird vocalization data preprocessing.

Due to the variety of audio file formats, audio format conversion is necessary. This study uses FFmpeg for audio format conversion to ensure uniform processing and analysis. Additionally, to improve signal clarity, spectral subtraction is used for noise reduction. For segments with no sound, endpoint detection is performed to ensure that only effective bird vocalization segments are extracted. Finally, after completing these preprocessing steps, feature extraction can be performed on the audio data, providing effective features for subsequent recognition models. Fig 3 shows the entire data preprocessing process.

#### Subtraction denoising of bird vocalization.

This study selected spectral subtraction for denoising [22]. The recording of bird vocalization usually contains a variety of noises, such as wind, rain, and other environmental background noises. These noises will affect the clarity of bird vocalization and interfere with subsequent analysis and recognition tasks. Spectral subtraction is a classical denoising method. It is based on the characteristic difference between noise and signal in the frequency domain. It restores the pure signal by estimating the noise spectrum and subtracting the noise part from the total spectrum. It is suitable for stable background noise, such as uniform wind noise or constant mechanical noise. However, spectral subtraction has limitations in dealing with non-stationary noise, which may lead to distortion of speech or bird vocalization, and even introduce music noise, affecting the naturalness of sound. In order to improve the denoising effect, the Adaptive Averaging Exponent is introduced, which can dynamically adjust the denoising intensity to adapt to different types and different time-varying noises. Through this improvement, spectral subtraction can not only remove non-stationary noise more effectively, but also reduce signal distortion and make bird vocalization clearer, thus improving the accuracy of subsequent analysis.

$${\left|\hat{X}(\omega )\right|}^{p}={\left|Y(\omega )\right|}^{p}-{\left|\hat{D}(\omega )\right|}^{p}$$ (1)

Where |Y(ω)| represents the magnitude spectrum, $\hat{D}(\omega )$ is the estimated noise magnitude spectrum, and p is the power exponent. When p=1, it is amplitude spectrum subtraction; when p=2, it is power spectrum subtraction. Spectral subtraction is simple to implement and computationally efficient, suitable for environments with relatively stable background noise levels.

#### Bird vocalization endpoint detection.

In this study, Endpoint detection is based on short-time energy and zero-crossing rate.

Short-time energy is the total energy of the audio signal within a certain time window [23], typically used to identify voice segments as they usually have higher energy than silent segments. Short-time energy can be calculated by summing the squared values of samples in each frame:

$$E_{t}=\sum_{n=0}^{N-1}|x[n]{|}^{2}$$ (2)

Where x[n] is the amplitude of the n-th sample in time window t, and N is the number of samples in the frame. The zero-crossing rate is the number of times the audio signal changes sign within a unit time [23–25]. This feature captures the frequency characteristics of the voice signal.

$$Z_{t}=\frac{1}{2}\sum_{n=1}^{N-1}|sgn(x[n])-sgn(x[n-1])|$$ (3)

Where sgn(x) is the sign function, returning 1 if x[n] is greater than 0, and -1 if less than 0. N is the number of samples in the frame.

Threshold Setting: First, normalize the short-time energy and zero-crossing rate of each frame and calculate the weighted average. Then, calculate the mean energy and standard deviation of all frames, setting the threshold as:

Threshold=Mean+0.5*Standard Deviation (4)

This threshold determines whether each frame of audio contains valid speech information. If the energy or zero-crossing rate of the frame is higher than this threshold, it is considered to be a voiced frame; otherwise, it is considered to be a silent fragment.

#### Data augmentation.

In this study, a few birds had a low number of audio recordings at a certain time, such as Cyanocitta cristata, which had only 14 audio recordings at night. In this study, we used Time Stretching and Time Shifting as data augmentation methods. Audio stretching can adjust the time scale but maintain the pitch, while audio translation does not change the audio characteristics, only adjust the starting time point. In order to avoid impact on the audio features, we chose the stretching range of 0.8-1.2 times the speed, and the translation range of 0-100ms. These methods help to improve the robustness of the model to time changes while ensuring the diversity of data. The extended data is the audio of Cyanocitta cristata and Passerina cyanea at night, so that the total audio length is not less than 20 minutes.

Audio Stretching: Let x(t) be the original audio signal, and *α* be the stretching factor. The transformed audio y(t) can be represented as:

$$y(t)=x\left(\frac{t}{\alpha }\right)$$ (5)

If α>1, the audio slows down (decelerates); If α<1, the audio speeds up (accelerates).

Audio Shifting: Let *τ* be the time offset. The transformed audio y(t) can be represented as:

y(t)=x(t−τ) (6)

If τ>0, the audio shifts backward (delayed start); If τ<0, the audio shifts forward (advanced start). If 𝐭−τ falls outside the original audio time range, y(t) can be set to 0 (silence) or filled as needed.

### Second-order MFCC feature extraction

Mel-frequency cepstral coefficients (MFCC) [26] are widely used features in speech and audio processing, particularly in speech recognition and music information retrieval. In this study, second-order ($\Delta \Delta \hat{MFCC}$) difference coefficients were used to extract key information from the speech signal for subsequent recognition processing.

**Fig 4:**
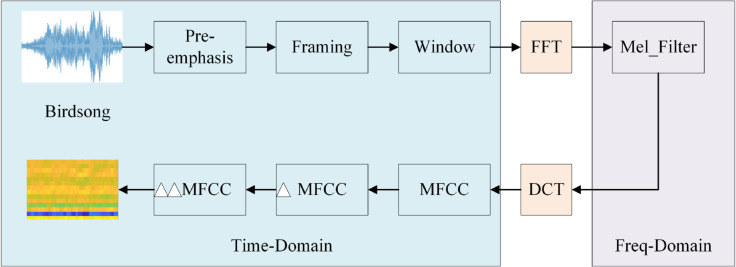
MFCC feature extraction.

Second-order MFCC captures the temporal dynamics of audio signals more finely, distinguishing sounds with similar spectral characteristics but different temporal features. This provides richer information than traditional MFCC and significantly enhances sound recognition accuracy and robustness. The process of extracting Second-order MFCC features is shown in Fig 4:

The formula for extracting MFCC features is:

$$|C_{n}=\sum_{M=0}^{M-1}\log\left(E_{m}\right).\cos\left[\frac{n(m+0.5)\pi }{M}\right]$$ (7)

To capture the temporal characteristics of the speech signal more comprehensively, in addition to the basic MFCC, second-order ($\Delta \Delta \hat{MFCC}$) difference coefficients were calculated.

Second-order Difference ($\Delta \Delta \hat{MFCC}$):

$$\Delta \Delta C_{t}=\frac{\sum_{n=-N}^{N}n.\Delta C_{t+n}}{2\sum_{n=1}^{N}n^{2}}$$ (8)

Where N typically takes the value of 1 or 2, effectively describing the trend of MFCC coefficients over time.

### Fine-grained recognition model of two layer deep network

In this study, the traditional machine learning network was used to identify the inter-species of the processed MFCC spectrogram. Then, on this basis, deep learning recognition models such as ResNet18 are introduced for fine-grained recognition. The core idea is to solve the problem of gradient disappearance and gradient explosion in deep neural networks by introducing residual blocks and jump connections, so as to achieve more efficient network training.

**Fig 5:**
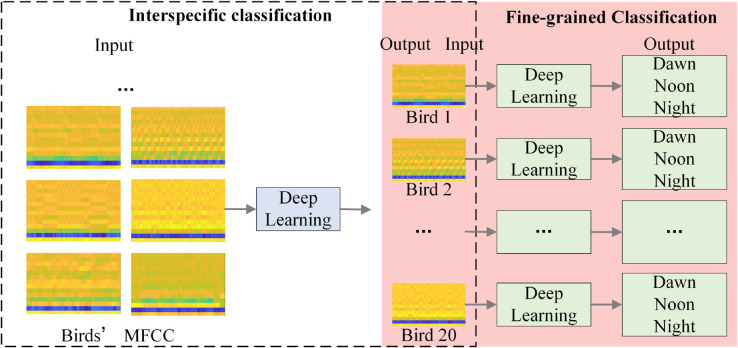
Fine-grained recognition framework for bird vocalization using two-layer deep network.

As shown in Fig 5, this process involves the use of ResNet18 for interspecific recognition, and then fine-grained recognition of bird vocalizations at different times of the day.

### Log-Mel ACI

The traditional ACI was proposed by Pieretti et al. in 2011 [8], and its core idea is to characterize acoustic complexity by quantifying the time intensity changes of acoustic signals. The formula is as follows :

$$ACI=\frac{D}{\sum_{k=1}^{n}I_{k}}$$ (9)

Where D is the difference sum of all frequency bins in a single time window, reflecting the acoustic complexity. *I*_*k*_ is the sound intensity value of a frequency bin in the k-th time step.

Traditional ACI calculates acoustic complexity based on the intensity change of linear spectrum, but the frequency distribution of bird vocalization is closer to the Mel scale of human auditory perception. The Mel filter bank can more accurately extract the frequency domain characteristics of bird vocalization by simulating the characteristics of human ears that are sensitive to low frequency and high frequency compression. In addition, the traditional ACI is not sensitive to the dynamic changes of low-intensity sounds, while the Log-Mel transform can enhance the ability to capture weak signals by compressing the dynamic range, which is more suitable for refined day-night acoustic pattern analysis. Log-Mel ACI is first passed through the Mel filter, but the logarithm is processed in the year, and finally calculated. The calculation process is as follows:

Step 1: Spectrogram Transformation. Transform the audio signal to the frequency domain using short-time Fourier transform (STFT), obtaining a complex spectrum. Calculate the magnitude:

$$Magnitude=\sqrt{{Real}^{2}+{Imaginary}^{2}}$$ (10)

Step 2: The Mel spectrum is closer to human auditory perception, and the Mel filter is used to convert the linear frequency to the Mel frequency : the Mel filter bank (20 triangular filters) is used to map the linear frequency to the Mel scale. This step simulates the characteristics of the auditory system, highlights the main frequency bands of bird vocalization (such as 1-1kHz), and suppresses irrelevant high-frequency noise:

Mel-spectrum=Mel Filter*Magnitude Spectrum (11)

Step 3: Logarithmic Scaling. Apply a logarithmic function to the Mel-spectrum to balance its dynamic range:

Log-Mel=log(1+Mel-spectrum) (12)

Where Log-Mel is the logarithm of Mel-spectrum.

Step 4: The Mel-spectrum difference of adjacent frames is calculated along the time axis, and the time variation law is analyzed. The time fluctuation intensity of the spectrum is quantified, which reflects the rhythm change of bird vocalization (such as syllable interval, call rate):

$$d_{k}(t)=|\text{Log-Mel}(k,t+1)-\text{Log-Mel}(k,t)|$$ (13)

where k is the Mel frequency index (= 1,2,..., 20), corresponding to the k-th triangular filter in the Mel filter bank ; t is the time frame index, which represents the t-th analysis window in the short-time Fourier transform (STFT). Log-Mel(k,t) represents the logarithmic Mel-spectrum value at the time frame t and Mel frequency k; $d_{k}(t)$ refers to the absolute difference value of the spectrum of adjacent time frames (t and t+1) at the Mel frequency k, which quantifies the time dynamic change of the frequency band.

Step 5: Log-Mel ACI Calculation. For each time frame, calculate the ratio of the differential $d_{k}(t)$ to its corresponding Mel-spectrum sum:

$$ACI_{Log\text{-}mel}=\frac{\sum_{k}d_{k}(t)}{\sum_{k}Log\text{-}Mel(k,t)}$$ (14)

Where ${ACI}_{Log\text{-}mel}$ is the Log-Mel ACI of each time frame; $\sum_{k}d_{k}(t)$ represents the sum of time difference of all Mel frequency bands, which characterizes the dynamic complexity of acoustic signals ; $\sum_{k}\text{Log-Mel(k,t)}$ represents the sum of the logarithmic energy of the Mel frequency band, and the ratio of the normalized dynamic change to the overall energy.

### Evaluation metrics

The dataset was divided into a training set and a validation set at a ratio of 8:2, with Epoch set to 50 and Batch Size to 32. During training, the cross-entropy loss function and AdamW optimizer were used, with a learning rate of 0.001. To prevent overfitting, early stopping mechanism and data augmentation techniques were introduced during training. Finally, the model’s performance was evaluated on the validation set using accuracy, precision, recall, and F1 score. Table 2 explains the parameters used in the evaluation metric calculations

**Table 2:** Explanation of parameters in evaluation metric calculations.

Term	Description	Explanation
*TP*	True positive	Predicted as positive and actually positive
*FP*	False positive	Predicted as positive but actually negative
*FN*	False negative	Predicted as negative but actually positive
*TN*	True negative	Predicted as negative and actually negative

Accuracy is used to measure the performance of recognition models:

$$Accuracy=\frac{TP+TN}{TP+FP+FN+TN}$$ (15)

Precision is judged based on the prediction results:

$$Precision\;=\frac{TP}{TP+FP}$$ (16)

Recall is judged based on the actual samples:

$$Recall=\frac{TP}{TP+FN}$$ (17)

F1 score is the harmonic mean of precision and recall, used to balance these two metrics. The expression is:

$$F1=2*\frac{Precision*Recall}{Precision+Recall}$$ (18)

In order to verify the feasibility of multi-class bird vocalization recognition, an inter-species recognition experiment was first carried out. The second-order MFCC spectra of 20 bird species were input into the recognition model for training. Then, the audio data of a single bird species were selected and divided into three time periods: dawn, noon and night. The experimental process is similar to the multi-class recognition experiment, but the number of neurons in the output layer is adjusted to 3. This study lays a foundation for the subsequent ACI analysis of acoustic indicators.

## Results and discussion

### Inter-species recognition results

**Fig 6:**
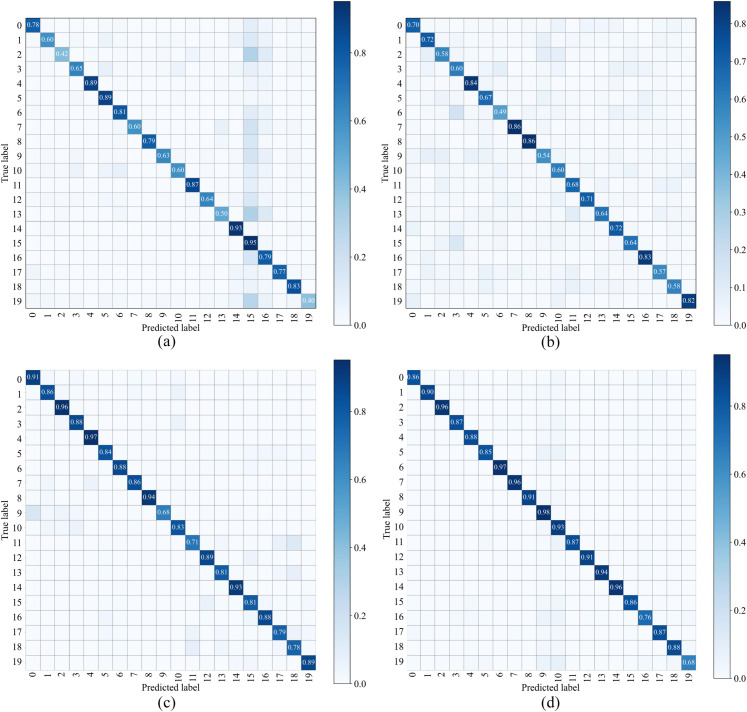
Confusion matrix of four types of networks. (a). KNN; (b). (SVM); (c). CNN+LSTM; (d). Resnet18

In the inter-species recognition of bird vocalization, both machine learning-based methods and deep learning-based methods were used. Experiments have demonstrated that the sounds of different birds can be identified using either approach. Among these methods, the ResNet18 model yielded the highest recognition accuracy of 95.65% for 20 bird species. This indicates that deep learning, particularly ResNet18, outperforms machine learning methods in bird vocalization recognition tasks. Table 3 presents the recognition results of the 20 bird species using the different recognition methods. Fig 6 is the confusion matrix of each recognition method. It can be seen from the figure that CNN + LSTM and Resnet18 perform better than KNN and SVM.

**Table 3:** Recognition results of 20 bird species.

Model	Accuracy	Precision	Recall	F1 score
*KNN*	0.8187	0.7999	0.7165	0.7453
*SVM*	0.7098	0.7086	0.7102	0.7072
*CNN* + *LSTM*	0.9521	0.9533	0.9341	0.9422
*ResNet*18	0.9565	0.9514	0.9381	0.9436

It can be seen from Table 3 that the recognition accuracy of KNN and SVM is 81.87% and 70.98% respectively, while the recognition accuracy of CNN + Resnet18 is 95.21% and 95.61% respectively. Similarly, as shown in Fig 6, the recognition accuracy of KNN and SVM for each category is basically below 80%, while the recognition accuracy of CNN + LSTM and Resnet18 for each category is basically above 80%. Compared with CNN + LSTM and Resnet18 models, Resnet18 is superior to CNN + LSTM. These results show that deep learning has great advantages in speech signal processing among machine learning and deep learning methods, which is consistent with the research results in 2021 [27]. Therefore, the Resnet18 model in deep learning is selected in the subsequent fine-grained recognition.

### Fine-grained recognition results

In the task of classifying a single bird species at different times of the day (dawn, noon, night), the ResNet18 model, which performs well in inter-specific recognition, was used. Table 4 shows the recognition results of 20 species of birds in the dawn, noon and night.

**Table 4:** Results of fine-grained recognition.

Bird Name	Family	Dawn	Noon	Night
*Cyanocittacristata*	0.9602	0.9651	0.9568	0.9592
*Pipiloerythrophthalmus*	0.9380	0.9396	0.9380	0.9384
*Cardueliscarduelis*	0.9367	0.9390	0.9361	0.9371
*Marecastrepera*	0.9588	0.9593	0.9588	0.9587
*Larusargentatus*	0.9056	0.9057	0.9047	0.9038
*Colaptesauratus*	0.9757	0.9760	0.9754	0.9755
*Agelaiusphoeniceus*	0.8665	0.8600	0.8623	0.8571
*Cardinaliscardinalis*	0.9553	0.9511	0.9520	0.9510
*Troglodytesaedon*	0.9427	0.9424	0.9427	0.9423
*Passerdomesticus*	0.9647	0.9650	0.9642	0.9644
*Zonotrichialeucophrys*	0.9605	0.9616	0.9607	0.9609
*Icterusgalbula*	0.9190	0.9055	0.9132	0.9082
*Certhiaamericana*	0.9583	0.9607	0.9583	0.9584
*Myiarchuscrinitus*	0.9421	0.9543	0.9404	0.9458
*Laniusexcubitor*	0.9766	0.9727	0.9711	0.9710
*Passerinacyanea*	0.9294	0.9008	0.8981	0.8963
*Seiurusaurocapilla*	0.9780	0.9791	0.9780	0.9781
*Haemorhouspurpureus*	0.9583	0.9586	0.9583	0.9584
*Pirangarubra*	0.9491	0.9508	0.9491	0.9498
*Contopussordidulus*	0.9468	0.9398	0.9473	0.9427

It can be seen from Table 4 that the recognition accuracy of the selected 20 species of birds is very high. This verifies that the song of a single bird at different times of the day is different and can be accurately identified in deep learning [4]. Among them, Seiurus aurocapilla had the highest recognition rate of 97.80%, and Agelaius phoeniceus had the lowest recognition rate of 86.65%, which may be due to other noise and bird interference. Therefore, in the follow-up study, it is also necessary to find a solution to the noise interference of complex bird vocalization.

### Recognition performance of MFCC

**Fig 7:**
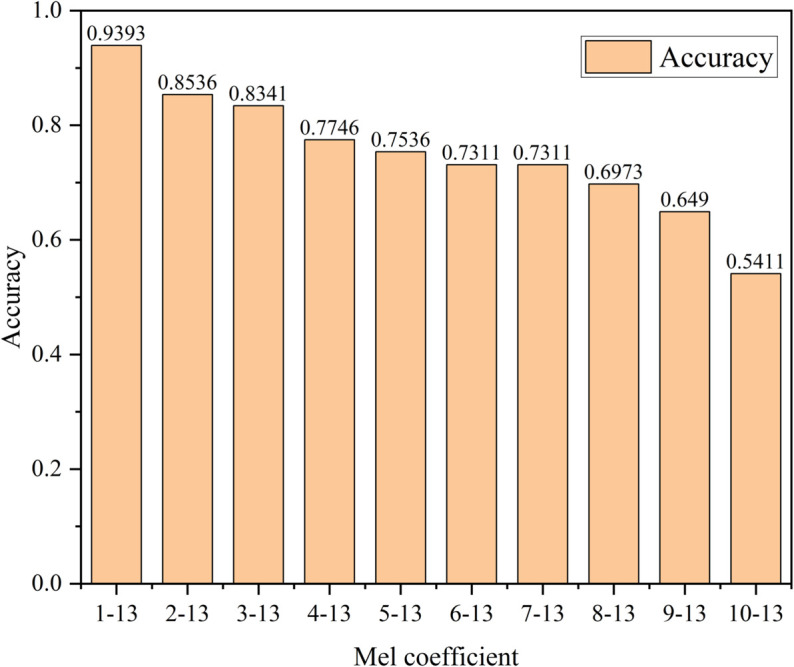
MFCC contribution analysis.

In this study, the second-order MFCC spectrum after Log-Mel transform was used for interspecific recognition and fine-grained recognition. Fig 7 analyzed the contribution of 13-dimensional MFCC coefficients. In each recognition, one of the 13 dimensions was reduced in turn, such as 2-13, which reduced the first dimension, and 3-13 reduced the first and second dimensions.

**Fig 8:**
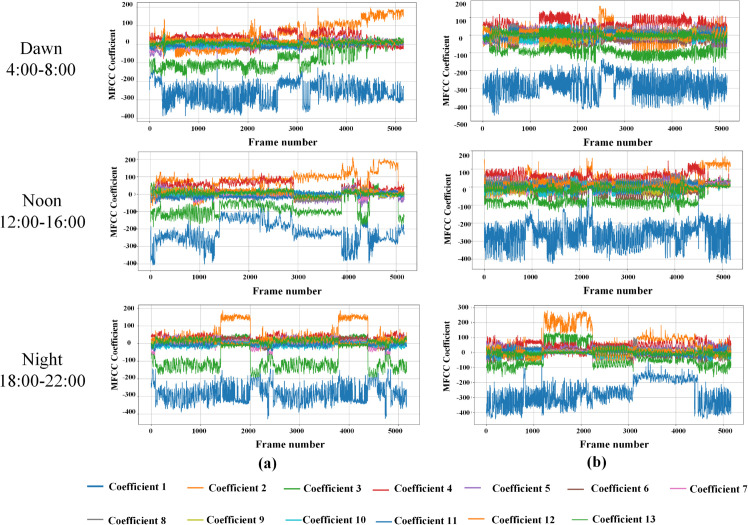
MFCC coefficient analysis of different kinds of bird vocalization at dawn, noon and night. (a). Cyanocitta cristata; (b). Pipilo erythrophthalmus

In addition, two species of 20 bird species were randomly selected, and the variation of MFCC coefficient of each bird with time was analyzed. The results were shown in Fig 8. The figure showed the variation curves of MFCC coefficients of these two birds at dawn, noon and night.

The experimental results show that the second-order MFCC not only performs well in interspecific recognition, but also performs well in fine-grained recognition [28] It can be seen from Fig 7 that the recognition accuracy is 93.93% when the 13-dimensional MFCC coefficients exist. After each reduction of one dimension, the recognition accuracy decreases. When the MFCC coefficient is reduced to only 4 dimensions, the recognition accuracy is only 54.11%. This shows that each dimension plays a vital role in the 13-dimensional MFCC coefficients.

In addition, this study also analyzed the performance of 13MFCC coefficient in different time periods of each bird, as shown in Fig 8. It can be seen from the figure that the MFCC coefficients of the same bird vocalization are different at different times of the day. This showed that the bird vocalization at dawn, noon and evening were different. This difference can be reflected not only in deep learning, but also in ecological index.

### Analysis of diurnal bird vocalization patterns based on Log-Mel ACI

**Fig 9:**
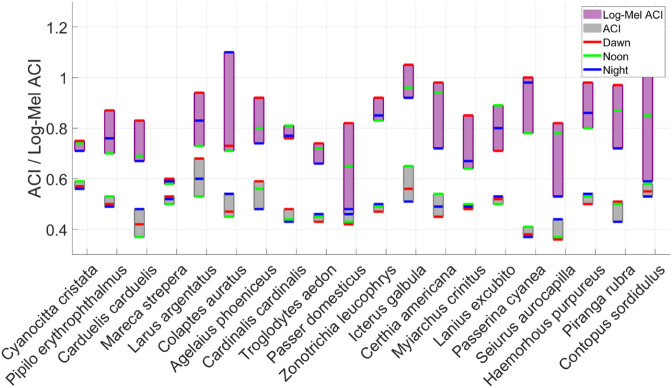
Different calculation results of 20 kinds of bird vocalization based on traditional ACI and Log-Mel ACI are compared.

In this paper, the acoustic index calculation method of Log-Mel ACI is proposed and compared with the traditional ACI calculation, as shown in Fig 9. Grey represents the traditional ACI, and purple represents Log-Mel ACI.

It can be seen from Fig 9 that the traditional ACI calculation of 20 kinds of bird vocalization does not change much at different times of the day. Among the 20 kinds of ACI calculations, only 8 kinds of dawn are greater than noon, lacking difference and regularity. On the contrary, our Log-Mel ACI expands the difference between dawn, noon and dusk. For example, Passer domesticus and Contopus sordidulus, the difference between different times increased after using the acoustic index Log-Mel ACI. In addition, it can be seen from the figure. Among the 20 categories, there are 18 categories of Log-Mel ACI dawn is greater than noon, only Cardinalis cardinalis and Lanius excubito were larger than dawn at noon.

Studies have shown that Log-Mel ACI can be used as a robust indicator to reflect the activity of birds at different times of the day. Among them, Log-Mel ACI at dawn is usually the highest, indicating that dawn birds are more active, which is usually related to territorial declaration or courtship activities. The Log-Mel ACI value is generally the lowest at noon, which may be affected by high temperature or weather, and the decrease of bird predation at noon leads to the decrease of bird vocalization activity. At night, the values of Log-Mel ACI of 20 species of birds are generally lower than those at dawn, and some birds will have higher values than those at noon. It shows that the social activities of birds at night have recovered. This study shows that Log-Mel ACI effectively captures the circadian rhythm differences of bird vocalization.

### Innovations and limitations

In this study, a two-layer deep recognition network is proposed. Based on the second-order MFCC spectrum after Log-Mel transformation, not only the inter-species recognition of bird vocalizations can be performed, but also the dawn, noon and evening sounds of a single species can be fine-grained identified. In addition, a novel Log-Mel ACI calculation method is proposed, which has obvious advantages over traditional ACI. This study also successfully built a bridge for the application of deep learning technology and acoustic indicators through Log-Mel, demonstrating the potential of advanced data analysis methods in complex ecosystems [29]. Through this innovative approach, we can better understand the dynamic changes of bird ecosystems and provide new insights and methods for future ecological research and environmental protection. In summary, the fine-grained identification and Log-Mel ACI calculation in this study have broad application prospects in bird ecology research.

However, this study still has several limitations for improvement. Firstly, in this study, some of the 20 birds selected failed to completely remove the noise, resulting in lower recognition accuracy than other birds. Therefore, in complex environments, this study has not yet found an ideal denoising method. Future research will focus on improving denoising algorithms and introducing stronger non-stationary noise suppression techniques to more effectively deal with noise problems in complex environments, thereby improving the recognition accuracy of all bird species. Secondly, the limited types of data sets selected in this study may have an impact on the broad applicability of the method. Therefore, future research should focus on expanding the diversity of data sets to cover more birds and different environmental conditions, and enhance the generalization ability of the model. In addition, future research should also apply Log-Mel to other acoustic indices, such as Bioacoustic Index (BI) and Acoustic Diversity Index (ADI), to explore the applicability and effectiveness of Log-Mel in a variety of ecological acoustic indicators.

## Conclusion

In this study, the fine-grained recognition network and Log-Mel ACI acoustic index analysis were used to explore the song patterns of birds in different time periods from two complementary perspectives. The results show that the second-order MFCC spectrum and recognition model based on Log-Mel can effectively distinguish the birdsong in different time periods, and the recognition accuracy is about 95%. In addition, the improved Log-Mel ACI acoustic index calculation clearly shows the time law of bird vocalization : the highest at dawn, followed by night, and the lowest at noon. These results suggest that the combination of fine-grained identification (classification) and acoustic index calculation (ecological analysis) can jointly reveal the variation of the same bird ’s vocalization in different time periods. In addition, this study combines deep learning technology and acoustic indicators, providing new tools and methods for bird ecology research, and providing a new perspective for the analysis and understanding of bird behavior patterns. This method not only improves the classification accuracy of bird song, but also reveals its acoustic characteristics in different time periods, which provides strong data support for ecological monitoring. This study pioneers the application of ACI to single vocalizations, rather than extended soundscapes, enabling fine-grained analysis of circadian vocalization rhythms. The proposed Log-Mel ACI demonstrates improved temporal sensitivity and may open new directions for ecoacoustic monitoring. In addition, compared with other ecological acoustic indices, ACI combined with Log-Mel transform can better capture biological acoustic features, making it an important tool in ecological research.
